# Household related predictors of burn injuries in an Iranian population: a case–control study

**DOI:** 10.1186/1471-2458-12-340

**Published:** 2012-05-09

**Authors:** Homayoun Sadeghi-Bazargani, Shahnam Arshi, Mehrnaz Mashoufi, Reza Deljavan-anvari, Mohammad Meshkini, Reza Mohammadi

**Affiliations:** 1 Medical Philosophy and History Research Center, Statistics & Epidemiology Department, Tabriz University of medical sciences, Tabriz, Iran; 2 Department of public health sciences, Shahid Beheshti University of medical sciences, Tehran, Iran; 3 Ardabil University of medical sciences, Ardabil, Iran; 4 Injury epidemiology and prevention research center, Tabriz University of medical sciences, Tabriz, Iran; 5 Medical student, Injury epidemiology and prevention research center, Tabriz University of medical sciences, Tabriz, Iran; 6 PHS Department, Karolinska Institute, Sweden. Injury epidemiology and prevention research center, Tabriz University of medical sciences, Tabriz, Iran

**Keywords:** Burns, Injuries, Risk factors, Epidemiology, Predictors, Case- control studies, Cooking, Stove, Iran

## Abstract

**Background:**

To prevent burn injuries it is vital to have sound information on predictors of its occurrence in different settings. Ardabil Province is the coldest province of Iran with high burden of burn injuries. The aim of this study was to determine the household related predictors of unintentional burns in Ardabil Province located at North-West of Iran.

**Methods:**

The study was conducted through a hospital based case–control design. 239 burn victims as well as 246 hospital-based controls were enrolled. Both bivariate and multivariate analysis methods were used.

**Results:**

Males comprised 55.2% of all the study subjects. Mean age of the participants was 21.8 years (95% CI: 19.17-24.4). The economic ability of the households was associated with risk of burn injuries. Multivariate conditional logistic regression results showed the following variables to be independent factors associated with burn injuries. Using non-conventional pipe-less air heaters instead of conventional piped kerosene- or gas-burning heaters (Odds ratio: 1.98, 95% CI: 1.1-3.6). Common use of picnic gas-stove for cooking at home (odds ratio = 1.6, 95%CI: 1–2.4). Using electric samovars instead of other types of samovars (Odds ratio = 0.3, 95% CI: 0.1-1). Using samovars lacking the national standard authorization mark (Odds ratio = 2.2, 95% CI: 1.4-3.6).

**Conclusion:**

Using some types of specific heating or cooking appliances, and unsafe use of conventional appliances were major risk predictors of burn injuries in this population.

## Background

Burns are considered as an important cause of mortality in low and middle income countries [1,2]. About 90% of burn deaths occur in these countries, where prevention programs are uncommon and the quality of acute care is inconsistent [3]. It is estimated that 3-4% of burnt patients need to be admitted in specialty centers, and about 25,000 people die due to burns annually [1]. Young children especially those under six years of age are the most vulnerable ones for burn injuries. Hot water and liquids and open fire respectively are the most important causes of burns [2]. Injury epidemiology is defined as the study of the distribution and determinants of injuries and safety related states-events in specified populations, and the application of this study to prevent injuries and promote safety [4]. The approach to burn prevention, to be effective in a particular area, should be based on sound knowledge of etiological patterns of burn injuries. Some preventive measures have been shown to be quite effective in reducing burn injuries. Nevertheless, most of the evidence comes from high-income countries. This is while the patterns and risks of burns can be quite different in low and middle income countries (LMICs), and few of these interventions are readily transferable to LMICs [5-7]. Burns are also considered as a major public health problem in Iran and in other Eastern Mediterranean countries [8-10]. The possible risk factors or predictors of burn injury may be different in high income countries and LMICs. There may be some similarities as well as differences from country to country. Therefore, an abundant amount of information from different countries is needed to define the risk factors of burns in a reliable manner.

Available studies assessing the predictors of burn injuries especially from LMICs and eastern Mediterranean countries are quite limited [9,10]. Moreover, except for few studies, less attention is paid to assess safety of heating/cooking appliances as possible predictors of burn injuries.

The aim of this study was to determine the predictors of unintentional burns in Ardabil Province, North-west of Iran.

## Methods

Study was conducted within a period of 18 months during 2007–2008 in Fatemi burn center which is the provincial referral burn center in Ardabil Province in the North-West of Iran. This center receives burn injuries from the nine districts of Ardabil Province with a population of roughly 1200000 people. The study was conducted through a hospital-based case–control design. Patient enrollment was done prospectively, over a period of two years.

### Cases

Cases in this study were 239 burn victims hospitalized in Ardabil provincial burn center in Fatemi university hospital during the years 2007–2008. All inpatient burn victims were enrolled into this study whether they died after admission, were discharged or were transferred to the more specialized centers outside Ardabil Province.

### Controls

These were 246 hospital-based controls selected according to the available control selection principals for case–control studies [11-13]. They were hospitalized patients from the other wards of Fatemi University Hospital and Alavi University Hospital. Controls were selected on a basis to ensure common source populations for the cases and controls. Therefore, the wards that received patients only from the province capital or the nearby locations were excluded. For example obstetrics ward was exluded because its referral pattern was not similar to burns center. The following inclusion and exclusion criteria were adopted for the case group:

Inclusion criteria:

1- Patients of both sexes living in Ardabil Province

2- Patients with unintentional burn injuries admitted to Ardabil Burn Center

3- Patients with thermal burn injuries including scaldsflame burns, and contact burns.

4- Willing to participate in the study

Exclusion criteria:

1- Frostbites and chemical burns

2- Self-immolation and other intentional burns

3- Burn injuries occurred out of Ardabil Province

4- Outpatient admissions

The following inclusion and exclusion criteria were adopted for control group:

Inclusion criteria:

1- Patients living in Ardabil Province

2- Lacking a hsitoy of burn injuries during the month before enrollment

3- Admitted to one of the university hospital wards in Ardabil Province that share a common refference population with Ardabil Burn Center

4- Patients of the same age and sex as of the cases

5- Patients of the same urbanity status (rural vs. urban) as of the cases

6- Willing to participate in the study

Exclusion criteria:

1- Admitted to hospitalbecause of chronic diseases

2- Admitted to hospital because of other major types of injuries

3- Outpatient admissions

**Figure 1:**
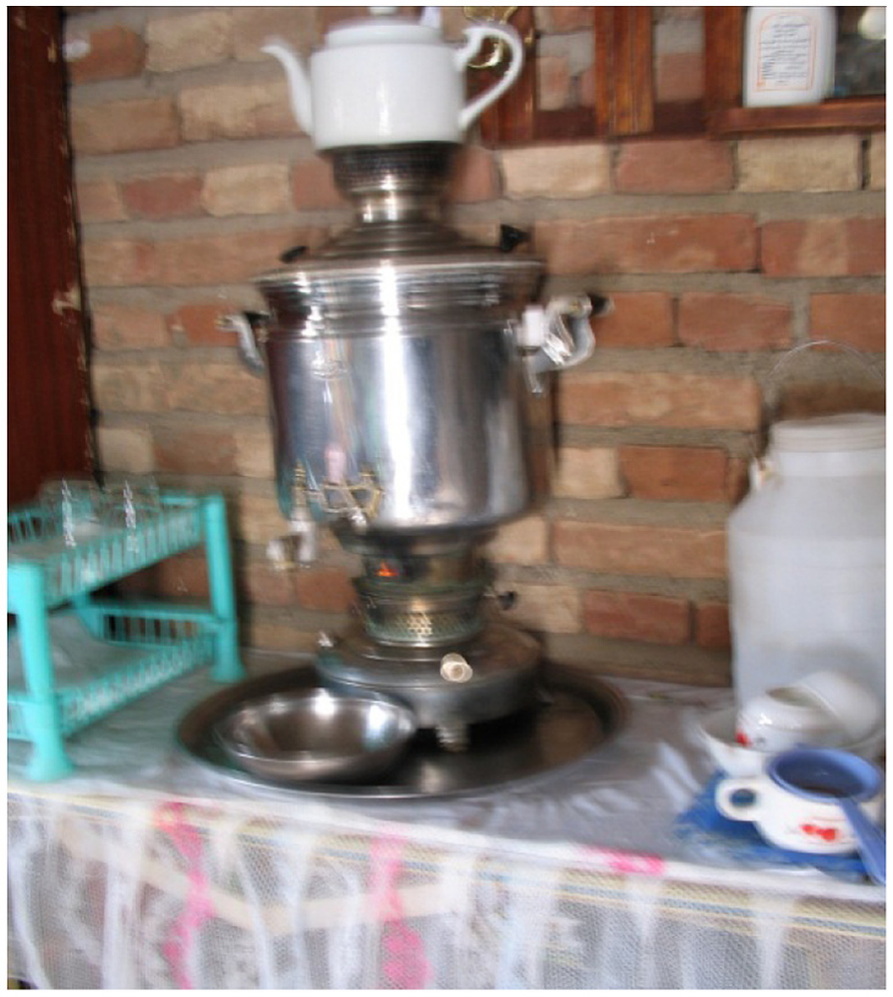
A samovar boiling water as well as holding a kettle on it to brew the tea.

Information regarding outcome, severity, extent of burn, and ICD 10 coding was retrieved from medical records. As the ICD coding is done after discharge from hospital, to increase the validity of information regarding injury outcome, this last stage in data collection took place after primary were collected by the questionnaire. The questionnaire was completed through interview except for some cases who asked voluntarily to fill in the questionnaire themselves. In such cases questionnaire was reviewed by the interviewer after being completed. Interviews were made with adult patients. In case of children and also few severe burn or disease cases, the caregivers were interviewed. Interviewers were chosen from the hospital’s medical registry staff who worked in shifts. This helped to capture interview chance for mortal cases early during their hospitalization. Mainly three medical registry experts conducted the interviews. These staff had completed a two-year long academic education in medical registry before being employed by the hospital. They participated in a short training session and did a supervised pilot data collection to ensure lower interviewer variability. A specific questionnaire was completed only for burn victims to assess patterns of injury occurrence from a prevention perspective, results of which are published elsewhere [14]. However, regarding the aims of this case–control study, the questionnaires were completed both for cases and control patients. Data were collected regarding patient history and demographics such as sex, age, and household economic level; house structure and decoration, safety status of cooking appliances, heating appliances, human knowledge, attitude and behaviors. All types of appliances including traditional heaters like samovars and valors were investigated. A samovar (Figure 1) is a heated metal container traditionally used to heat and boil water in and around Russia, as well as in other Slavic nations, Iran, Kashmir and Turkey [15].

Questions were based on tools provided through a joint Iran-Sweden research project on epidemiology of burns and a previous PhD thesis [7,16].

**Figure 2:**
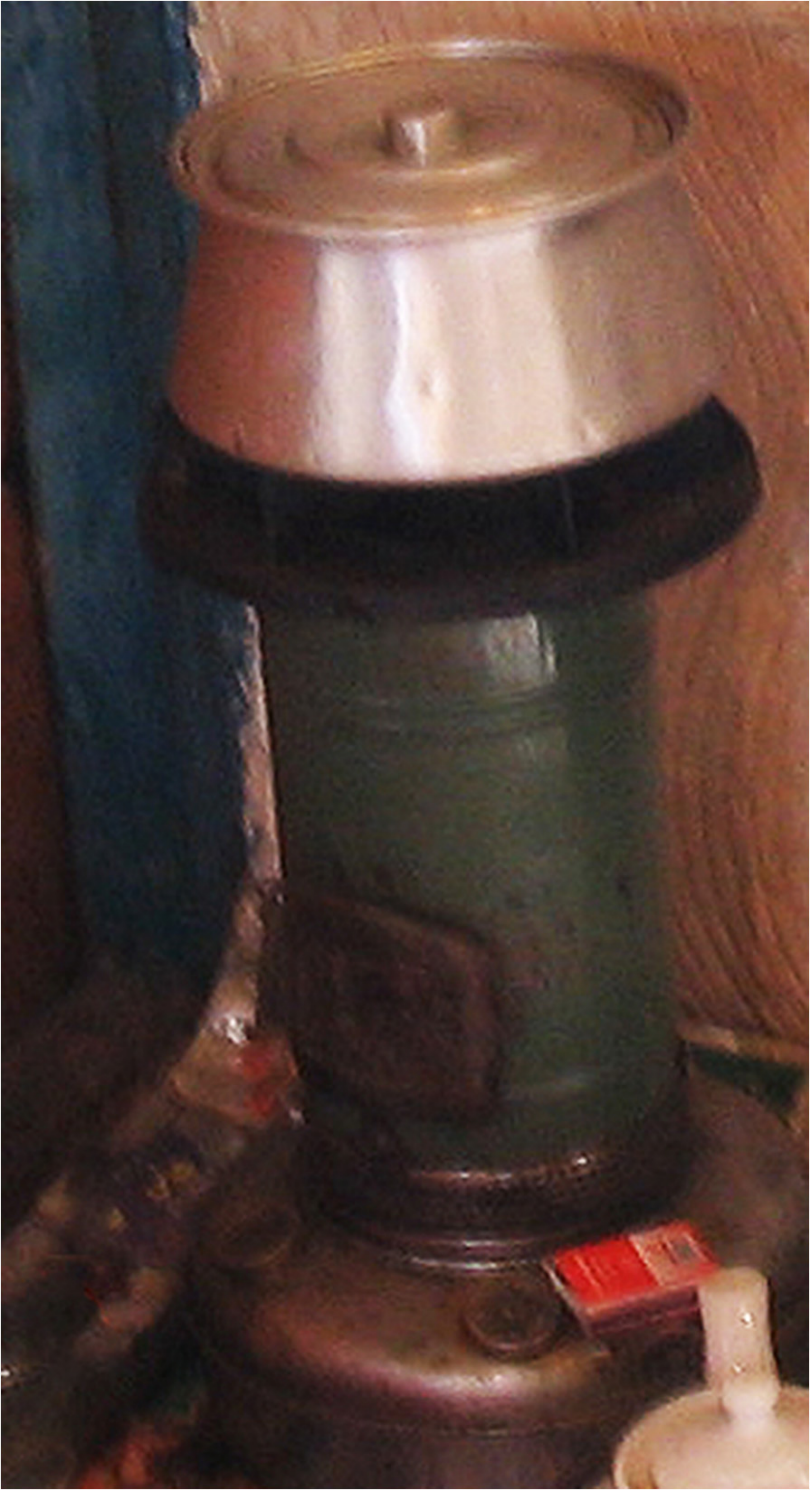
Valor, a traditional Iranian appliance, used both for cooking and making tea as well as heating the air.

A valor and, similar to it, an aladin is a traditional pipe less cooking appliance sometimes used for dual purpose of cooking and heating the air (Figure 2).

To provide necessary power of study over preventable risk factors, cases were matched with controls on age, sex and urbanity through frequency matching method. Statistical analyses were done using STATA statistical software package (Release 11. College station, TX: stata Corp LP.). Both bivariate and multivariate analysis methods were used. An independent samples t-test was used to compare the means of normally distributed numeric dependent variables for two independent groups. The Wilcoxon-Mann–Whitney test was used as a non-parametric analog to the independent samples t-test when the normality assumption didn’t hold. A One-Way Analysis of Variance was used to test the equality of three or more means. To assess the association of two categorical variables, such as study group and use of electric samovars, Chi-squared test was primarily applied. Fisher’s exact test was used if the expected count limit assumption was not met. Also crude odds ratios were calculated and their 95% confidence intervals were reported. Associations with a p-value < 0.1 were considered to be adjusted in multivariate conditional logistic regression analysis and the adjusted odds ratios along with their 95% confidence intervals were reported for the variables that were kept in the final model. Statistical significance was set at P < 0.05 (two tailed test results were applied).

All the eligible burn patients admitted to Ardabil Burn Center over a period of two years were enrolled and the study was powered to detect 10% difference in frequency of the occurrence of dichotomous predictors from a base control group relative frequency of 5-15% and accepting at most 0.05 type I and 0.1-0.3 type II errors.

### Ethical issues

The study protocol was approved by regional committee of ethics located in Ardabil University of medical sciences. Research was carried out in compliance with the Helsinki Declaration.

## Results

**Figure 3:**
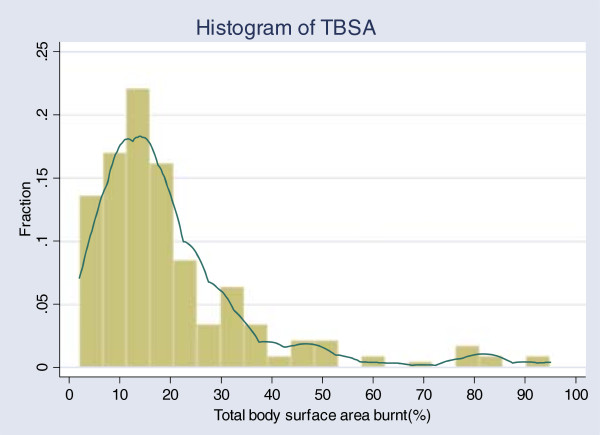
Histogram of the percentage of total body surface area (TBSA) burnt among burn victims.

Males comprised 55.2% of all the study subjects. Mean age of the participants was 21.8 years (95% CI: 19.17-24.4). More than 80% of burns occurred at home and kitchen was the most common place of injury. Nearly half of the burns were scalds. Median of total body surface area (TBSA) burnt was 15 percent with an inter-quartile range (IQR) of 10%. Histogram of TBSA distribution is given in Figure 3.

### Bivariate analysis results

Mean household size (number of household members) was 5.4 in case group versus 5.3 in control group, but without statistical significance. Mean roofed living area per member of the family was 25.8 square meters in case versus 25 m^2^ in control group without statistically significant difference. Slightly higher percentage of those living in houses without a separate kitchen belonged to case group(51% vs. 49%), but the difference was not statistically significant.

The economic ability of the households was associated with the risk of burn injuries, such that, having a very poor financial expenditure ability increased the chance of burn injury (OR = 1.7, 95% CI: 1.1-2.6). Consistent use of cooking gas stove as an air heating device in winter was associated with burn injuries(Fisher exact test : P < 0.01).

Fifty-eight percent of households who used kettles for boiling water to make tea belonged to case group, but using other facilities like samovars was more common among control group households. The P-value was borderline not significant.

Considering samovar type, using electric samovars compared to other samovar types seemed to be a preventive measure in burn injuries (OR = 0.27, 95% CI: 0.1-0.7). Having a samovar without national standard authorization mark increased the odds of getting burnt (OR = 1.9, 95% CI: 1.2-3.1).

Although those who were used to cook food on air-heaters instead of using cookware were more likely to be in case group, the difference was not statistically significant. Although those who boiled water on air-heaters had higher chance of burns, the difference was not statistically significant.

The odds ratio of getting burnt in those households that always used the picnic gas at home was 2.1 (95% CI: 1.1-3.9). The odd ratio of getting burnt in those households that always used the valor for cooking at home was 1.7 (95% CI: 1.1-2.8). Using traditional pipe-less Valor and Aladdin heating devices to heat the house increased burn injury chance with an odds ratio equal to 3.4 (95% CI: 1.4-8.2).

The household heads were asked whether, during the next six months, they were willing to spend some money to improve their home safety, and if so, how much they preferred to spend? The higher their preference to spend money, the lower was the likelihood of getting burnt. It was also checked whether people knew about the national standard authorization mark. The odds ratio of getting burnt in those who distinguished the national standard organization mark, as shown to them, was 0.7 (95% CI: 0.45-0.99).

### Multivariate analysis results

Multivariate logistic regression results showed the following variables to be independent factors associated with burn injuries. 1- Using non-conventional pipe-less air-heaters instead of conventional piped kerosene or gas burning heaters. 2- Common use of picnic gas-stove for cooking at home. 3- Using electric samovars instead of other types of samovars. 4- Using samovars lacking the national standard authorization mark. Table 1 presents both the crude and adjusted odds ratios derived from the logistic regression analysis.

**Table 1:** Crude and adjusted odds ratios for the predictors of burn injuries that were included in the final regression model

**Predictors in model**	**Adjusted odds ratio(95%CI)**	**Crude odds ratio(95%CI)**
Using electric samovars instead of other types of samovars	0.3(0.1-1)	0.27(0.1-0.7)
Common use of picnic gas-stove for cooking at home	1.6(1–2.4)	1.3(0.9-1.9)
Using non-conventional pipe-less air heaters instead of conventional piped kerosene or gas burning heaters	1.98(1.1-3.6)	2.2(1.4-3.7)
Using samovars lacking the national standard authorization mark	2.2(1.4-3.6)	2.2(1.4-3.4)

## Discussion

The main finding in this study was that the problems with appliances, both cooking and heating appliances, played a major role in causing the burns to happen. This includes safety problems in appliance product safety as well as appliance usage including; place of usage, process of usage and purpose of usage. Similarly with findings of this study, kitchen is stated to be the main place of domestic burns in most hospital based injuries [2,3,10,17]. However, this may not be the case in minor burns [18]. Nevertheless, unsafe heating/cooking appliances or unsafe use of them to be predictors of burns, doesn’t exclusively mean that such risks are only present in kitchens. As found in this study, using traditional pipe-less Valor and Aladdin heating devices to heat the house may increase the risk of burn injuries. The common pattern of using these appliances in Iran is such that during the winter they are placed in living room where they can be used for dual purpose of cooking and heating the air (Figure 2). This may increase the chance that people at home, especially kids, bump into them [14,19]. The chance may be higher when these appliances are placed in the middle of the room, where is much frequented by running/playing kids, especially at the times that kids are left less supervised. Mashreky et al. have found that the vast majority of burns take place during the first half of the day, from 9 am to 1 pm, when mothers are busy with their household chores [20]. Lack of supervision can be identified as a major determinant of childhood burns. In Bangladesh also some kerosene appliances are used for heating or cooking purposes. Consistently with our findings, a prior study in Bangladesh also revealed that using these appliances increases the likelihood of getting burned by three times [21].

In our study the use of electric samovars, rather than kettles and non- electric samovars, reduced the risk of burn injuries. Using flammable fuel for heating and cooking in non-industrialized countries and also the explosions related to industrial activities in developed countries are important factors that increase the risk of burns [22,23]. Moreover, it could be noticed in our study findings that several of the unsafe appliances were not working on electricity/gas networks incorporated in the house. The question would be that if this is because of the lack of these facilities, or these households still use the same appliances for decades because they did not need to be replaced yet? In Iran, all cities have complete coverage of electricity to households. In rural areas, however, all villages with more than 20 households have access to electricity network. However, possibly due to higher cost of electricity in Iran, there are many people who continue to use kerosene burning appliances or gas-burning appliances. Gas networking, especially in rural areas, is not as wide as electricity. This is both due to national limitation in extending the networking, household financial limitations to establish a gas network, and houses lacking minimum structural standards of establishing gas networking.

In this study it was found through bivariate analysis that, the economic ability of the households was associated with the risk of burn injuries, such that, having a very poor financial expenditure ability increased the chance of burn injury. The issue of poverty and burn risk is a significant fact well discussed in literature [24-26]. However, if studies gain success in depicting the mechanisms through which the poverty increases burn risk, it will be easier and more cost-effective for the policy makers to decrease such a risk. Physical environment including the heating/cooking appliances is an example addressed in present study.

In present study it was found that using a samovar without national standard authorization mark increased the odds of getting burnt. Two notices should be taken in this regard. First is the necessity to develop or operationalize national legislations in order to prevent the production, import and sales of unsafe cooking heating appliances. A mandatory requirement in this regard will be to improve and extend available standards for heating/cooking appliances. Secondly as consistent with findings of the present study, would be to increase peoples knowledge on distinguishing standard and safe cooking/heating products available in market and also to motivate them to buy the appliances authorized by national standard organizations.

Our study results showed that the conventional oil-burning or gas-burning heaters may appear safer and less risky than other types of heating appliances. One explanation for this can be that such appliances are usually more strictly controlled by national standard organization. Also such appliances are usually larger, heavier and are placed close to the walls and out of walking area.

Our study showed that the picnic gas stove is used for cooking by some Iranian families and this was associated with nearly twice higher risk of burn injuries. This was also consistent with the Bangladeshi study indicating three times more odds of getting burned for children living in families using such types of appliances [21]. A picnic gas stove is an appliance which is made for short term cooking in picnics and open spaces and isn’t suitable for routine use in indoor area. The higher risk of using this facility for indoor cooking purposes may be sought in following mechanisms such that; it is light and unstable so can easily be overturned especially with a cooking pot or kettle on it; it is mostly used in low income families coinciding with other risks of injuries; and as a final note that only the canister part of this facility has a national standard in Iran while the holder part is usually produced in an unsafe quality, sometimes causing connection gas leakage as well as unstable holding of dishes put on them.

Contrary to the Bangladeshi study as mentioned above, in present study the family size was not found to be a determinant of burn injuries. Several explanations can be presented in this regard such as; lower variation in household (family) size in Iran compared to Bangladesh, higher group similarity in family size due to matching done for the urbanity, the fact that family size may only be a determinant of childhood burns, and the lower role of the family size in Iran due to reasons like smaller households or different patterns of burns in Iran.

Using unsafe heating or cooking appliances, and unsafe use of them are major risk predictors of burn injuries in this population. Both active and passive approaches need to be considered to develop strategies for burn prevention. Improving product safety through legislations and standardization as well as improving customer behavior and product usage behaviors through safety education is recommended in this regard. Limitations and strengths:

A limitation of this study was that, in spite of a moderate sample size and two-year long census enrollment, the study was not large enough either to do subgroup analysis for the outcome; such as for scalds, flame, and contact burns; or to do subgroup analysis for the predictors such as for gender and age groups. Nevertheless, it doesn’t seem to jeopardize the main objective of study and provides better generalizability for the whole population and general prevention programs. The main strength of this study was that a wide range of possible burn injury predictors were measured and properly addressed with a focus on heating and cooking appliances.

## Conclusion

Using some types of specific heating or cooking appliances, and unsafe use of conventional appliances were major risk predictors of burn injuries in this population.

## Competing interests

The authors declare that they have no competing interests.

## Authors’ contributions

HSB contributed in design and conduction of the research, analyzed the data, and helped in writing the manuscript. AS & RM participated as supervisors through the whole research, and reviewed the manuscript. MM & MM contributed in conduct and interpretation of data and helped in writing the manuscript. RD contributed in data analysis and interpretation. He also prepared the primary draft of the manuscript. All authors read and approved the final manuscript

## Pre-publication history

The pre-publication history for this paper can be accessed here:

http://www.biomedcentral.com/1471-2458/12/340/prepub

## References

[B1] Baker SP O'Neill B Ginsburg MJ Li G The injury fact book 1992 2 Oxford University Press, New York

[B2] Forjuoh SN Burns in low- and middle-income countries: a review of available literature on descriptive epidemiology, risk factors, treatment, and prevention Burns 2006 32 529 537 10.1016/j.burns.2006.04.002 16777340

[B3] Peck MD Epidemiology of burns throughout the world. Part I: Distribution and risk factors Burns 2011 37 1087 1100 10.1016/j.burns.2011.06.005 21802856

[B4] Sadeghi-Bazargani H Injury epidemiology and publishing injury research J Inj Violence Res 2012 4 1 10.5249/jivr.v4i1.200 22015495 PMC3291280

[B5] Liao CC Rossignol AM Landmarks in burn prevention Burns 2000 26 422 434 10.1016/S0305-4179(00)00026-7 10812263

[B6] Mock C Quansah R Krishnan R rreola-Risa C Rivara F Strengthening the prevention and care of injuries worldwide Lancet 2004 363 2172 2179 10.1016/S0140-6736(04)16510-0 15220042

[B7] Sadeghi-Bazargani H Epidemiology and statistical modeling in burn injuries 2011 Karolinska Institute publications, Stockholm ISBN: 978-91-7557-204-9

[B8] Akbari ME Naghavi M Soori H Epidemiology of deaths from injuries in the Islamic Republic of Iran East Mediterr Health J 2006 12 382 390 17037707

[B9] Othman N Kendrick D Epidemiology of burn injuries in the East Mediterranean Region: a systematic review BMC Publ Health 2010 10 83 10.1186/1471-2458-10-83 PMC2841676 20170527

[B10] Sadeghi-Bazargani H Mohammadi R Epidemiology of burns in Iran during the last decade (2000–2010): review of literature and methodological considerations Burns 2011 38 319 329 22119445 10.1016/j.burns.2011.09.025

[B11] Wacholder S Silverman DT McLaughlin JK Mandel JS Selection of controls in case–control studies III. Design options. Am J Epidemiol 1992 135 1042 1050 10.1093/oxfordjournals.aje.a116398 1595690

[B12] Wacholder S Silverman DT McLaughlin JK Mandel JS Selection of controls in case–control studies II. Types of controls. Am J Epidemiol 1992 135 1029 1041 10.1093/oxfordjournals.aje.a116397 1595689

[B13] Wacholder S McLaughlin JK Silverman DT Mandel JS Selection of controls in case–control studies I. Principles. Am J Epidemiol 1992 135 1019 1028 10.1093/oxfordjournals.aje.a116396 1595688

[B14] Sadeghi BH Arshi S Ekman R Mohammadi R Prevention-oriented epidemiology of burns in Ardabil provincial burn centre, Iran Burns 2011 37 521 527 10.1016/j.burns.2010.09.013 21131133

[B15] Sadeghi-Bazargani H Mohammadi R Arshi S Svanstrom L Ekman R The risks of using samovars as the main tea-preparing facility in some Eastern countries Burns 2008 34 1149 1152 10.1016/j.burns.2008.01.023 18513878

[B16] Arshi S Ekman R Sadeghi-Bazargani H Mohammadi R Epidemiology of burns and prevention model for burn injuries with specific focus on childhood burns in rural areas of Ardabil province 2008 Report, Ref Type

[B17] Vilasco B Bondurand A Burns in Abidjan, Cote d'Ivoire Burns 1995 21 291 296 10.1016/0305-4179(94)00001-E 7662131

[B18] Sadeghi-Bazargani H Mohammadi R Svanstrom L Ekman R Arshi S Hekmat S Epidemiology of minor and moderate burns in rural Ardabil, Iran Burns 2010 36 933 937 10.1016/j.burns.2009.10.022 20171014

[B19] Arshi S Sadeghi-Bazargani H Mohammadi R Ekman R Hudson D Djafarzadeh H Prevention oriented epidemiologic study of accidental burns in rural areas of Ardabil, Iran Burns 2006 32 366 371 10.1016/j.burns.2005.10.026 16529866

[B20] Mashreky SR Rahman A Chowdhury SM Giashuddin S Svanstrom L Linnan M Consequences of childhood burn: findings from the largest community-based injury survey in Bangladesh Burns 2008 34 912 918 10.1016/j.burns.2008.05.002 18674863

[B21] Mashreky SR Rahman A Khan TF Svanstrom L Rahman F Determinants of childhood burns in rural Bangladesh: A nested case–control study Health Policy 2010 9 3 226 230 20202714 10.1016/j.healthpol.2010.02.004

[B22] Adamo C Esposito G Lissia M Vonella M Zagaria N Scuderi N Epidemiological data on burn injuries in Angola: a retrospective study of 7230 patients Burns 1995 21 536 538 10.1016/0305-4179(95)00038-D 8540983

[B23] De-Souza DA Marchesan WG Greene LJ Epidemiological data and mortality rate of patients hospitalized with burns in Brazil Burns 1998 24 433 438 10.1016/S0305-4179(98)00043-6 9725683

[B24] Atiyeh B Masellis A Conte C Optimizing burn treatment in developing low- and middle-income countries with limited health care resources (part 1) Ann Burns Fire Disasters 2009 22 121 125 21991167 PMC3188147

[B25] Edelman LS Cook LJ Saffle JR Burn injury in Utah: demographic and geographic risks J Burn Care Res 2010 31 375 384 10.1097/BCR.0b013e3181db51b0 20375697

[B26] Laflamme L Hasselberg M Burrows S 20 Years of Research on Socioeconomic Inequality and Children's-Unintentional Injuries Understanding the Cause-Specific Evidence at Hand Int J Pediatr 2010 2010 819687 PMID: 20706660 10.1155/2010/819687 20706660 PMC2913857

